# Sclerosing Angiomatoid Nodular Transformation of the Spleen: A Case Report Highlighting Diagnostic Challenges and the Role of Immunohistochemistry

**DOI:** 10.7759/cureus.89994

**Published:** 2025-08-13

**Authors:** Thanh Thao Nguyen, Mitsuaki Yoshida, Vu Dung, Motona Kumagai, Sohsuke Yamada

**Affiliations:** 1 Department of Pathology and Laboratory Medicine, Kanazawa Medical University, Ishikawa, JPN; 2 Department of Joint Surgery, Vietnam Military Medical University, Hanoi, VNM; 3 Department of Pathology, Kanazawa Medical University Hospital, Ishikawa, JPN; 4 Department of Pathology II, Kanazawa Medical University, Ishikawa, JPN

**Keywords:** immunohistochemistry, sclerosing angiomatoid nodular transformation, spleen, splenectomy, vascular tumors

## Abstract

Sclerosing angiomatoid nodular transformation (SANT) of the spleen is a rare, benign vascular lesion that poses significant diagnostic challenges owing to its nonspecific clinical and radiological features. We herein report the case of a 35-year-old male who presented with chronic left hypochondrial pain and was found to have a splenic mass with poor enhancement on computed tomography (CT) and an increased fluorodeoxyglucose uptake on positron emission tomography-CT. Total splenectomy was performed, and a histopathological examination revealed well-demarcated angiomatoid nodules with varying vascular channels, fibrosclerosis, and hemosiderin deposition. Immunohistochemistry revealed a characteristic tri-phenotypic vascular profile of CD34^+^/CD31^+^/CD8^−^ capillaries, CD8^+^/CD31^+^/CD34^−^ sinusoid-like vessels, and CD31^+^ venules. Additional investigations ruled out associations between Epstein-Barr virus (EBV) and IgG4-related diseases. This case underscores the critical role of histopathology and immunohistochemistry in accurately diagnosing SANT and differentiating it from other splenic vascular tumors. We also emphasize the importance of considering SANT in the differential diagnosis of splenic masses, even in younger patients presenting with persistent unexplained abdominal pain.

## Introduction

Sclerosing angiomatoid nodular transformation (SANT) is a rare benign vascular lesion that primarily affects the spleen. It most frequently occurs in middle-aged adults, at a mean age of approximately 48 years, and shows a female predominance, accounting for approximately 67% of cases [1]. Most patients are asymptomatic, with lesions typically discovered incidentally during imaging for unrelated conditions, although some may experience vague abdominal pain or flank discomfort [2-4]. The preoperative diagnosis of SANT remains challenging owing to its rarity and nonspecific imaging features. Although the characteristic “spoke-wheel” pattern may become more apparent in delayed imaging phases [5], these findings remain nonspecific. Therefore, a definitive diagnosis ultimately relies on a histopathological evaluation. SANT must be distinguished from other benign vascular splenic lesions, such as hemangiomas, hamartomas, and littoral cell angiomas, as well as malignant tumors, including angiosarcoma, lymphoma, and metastases [3,6]. Histologically, SANT is characterized by multiple angiomatoid nodules with three distinct types of vascular channels, and this architectural pattern closely mimics the normal red pulp of the spleen [7,8]. However, the exact pathogenesis of SANT remains unclear [2,8]. Whether SANT represents a reactive process or a true neoplasm remains debatable [9].

We herein report a rare case of SANT that was diagnosed and managed at our institution. This report highlights the diagnostic challenges encountered and underscores the pivotal role of histopathological and immunohistochemical analyses in achieving an accurate diagnosis of splenic SANT.

## Case presentation

A 35-year-old man with no significant medical, medication, or family history presented with a two-year history of intermittent left hypochondrial pain and a sensation of tension in the back that had worsened over the past four months.

**Figure 1:**
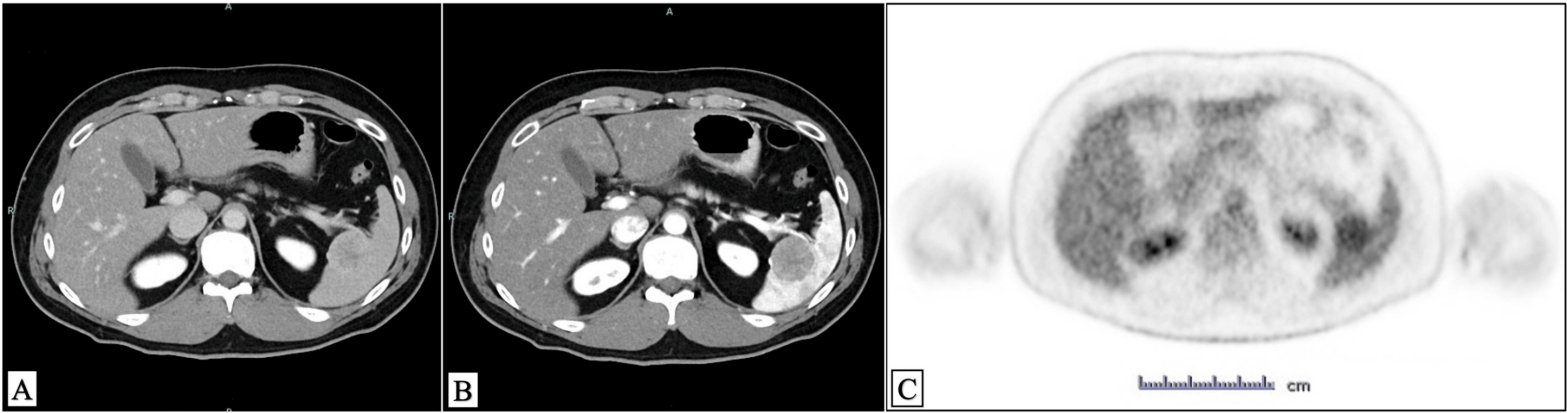
Radiological imaging (A, B) Contrast-enhanced computed tomography (CT) revealed a hypodense lesion in the spleen with relatively well-defined borders, measuring approximately 3 cm in diameter. In the early phase (A), the lesion showed low attenuation, while in the delayed phase (B), it demonstrated continuous internal arborizing enhancement. (C) Positron emission tomography (PET) demonstrated a solitary splenic mass measuring approximately 3 cm in diameter with a moderately increased fluorodeoxyglucose (FDG) uptake, heterogeneous in nature, and no evidence of distant metastasis.

No abnormalities were found in blood tests. Contrast-enhanced computed tomography (CT) revealed a splenic mass with poor enhancement in both early and delayed phases (Figure 1A, 1B). In positron emission tomography (PET)-CT images, a high fluorodeoxyglucose (FDG) uptake was observed in the same region of the spleen (Figure 1C). No distant metastasis was detected, and the patient underwent total splenectomy. The postoperative course was uneventful, with no acute complications observed. The patient was discharged early, remained stable, and was asymptomatic during follow-up visits. Scheduled surveillance was conducted at three, six, and 12 months postoperatively, followed by annual assessments to facilitate early detection of recurrence or late postoperative complications.

**Figure 2:**
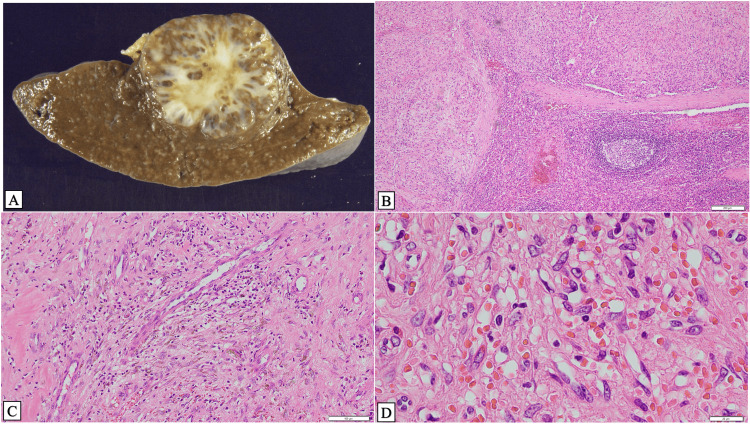
Gross and histological features (A) Gross specimen of the total splenectomy showing a spleen measuring 113 × 91 × 43 mm with a well-demarcated, white tumor measuring 37 × 31 mm, displaying an internal arborizing appearance. (B) Microscopic view showing the lesion located in the upper portion of the spleen, clearly demarcated from the surrounding normal tissue (located in the lower right side) by a thick fibrous capsule. Scale bar: 200 μm. (C) A histopathological examination showing proliferative growth of aberrant blood vessels with slit-like or irregular lumina, accompanied by hemosiderin deposition and interstitial fibrosis. Scale bar: 100 μm. (D) Abnormal small-sized blood vessels arranged in clusters. Aggregates of lymphoid cells are also observed. Scale bar: 20 μm.

Regarding pathological findings, a gross examination revealed a spleen measuring 113 × 91 × 43 mm and weighing 165 g after fixation. At the splenic hilum, a whitish nodule measuring 37 × 31 mm with clear borders and an internal arborizing structure was observed (Figure 2A). Histologically, the lesion was clearly delineated from the surrounding splenic parenchyma using a well-defined fibrous capsule (Figure 2B). Within the lesion, proliferation of abnormal vascular channels with a slit-like, irregular morphology was observed, accompanied by stromal fibrosis and hemosiderin deposition (Figure 2C). Vascular structures became smaller and aggregated into clusters, accompanied by the presence of lymphoid aggregates (Figure 2D).

**Figure 3:**
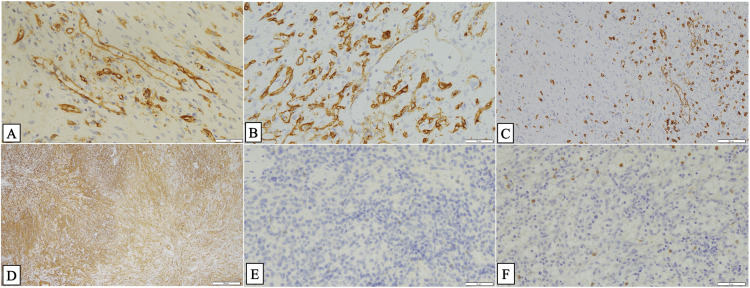
Immunohistochemical and molecular findings (A) Immunohistochemical staining for CD31 showing positive expression in the walls of small blood vessels. Scale bar: 50 μm. (B) CD34 staining showing positive expression in the walls of capillaries but negative staining in sinusoidal vessels. Scale bar: 50 μm. (C) CD8 immunostaining showing positive expression in the walls of sinusoidal vessels but negative staining in the walls of capillaries. Scale bar: 100 μm. (D) Alpha-smooth muscle actin (α-SMA) staining was diffusely positive, although the distribution was uneven with thick and sparse areas. Scale bar: 500 μm. (E) In situ hybridization for Epstein-Barr virus-encoded RNA (ISH-EBER) in the splenic lesion showed a negative result. Scale bar: 50 μm. (F) IgG4 immunostaining revealed only a few scattered IgG4-positive plasma cells with very low density, suggesting an IgG4-negative or weakly positive result. Scale bar: 50 μm.

Immunohistochemical staining showed positive for CD31 and CD34 in capillaries, while CD8 was positive in sinusoidal vessels and negative in capillaries (Figure 3A-3C). Alpha-smooth muscle actin (α-SMA) showed diffuse positivity, with both crowded and sparse areas of staining (Figure 3D). Based on these histological and immunophenotypic features, a diagnosis of SANT of the spleen was established. Additional investigations, including Epstein-Barr virus (EBV)-encoded RNA in situ hybridization (ISH-EBER) in the nuclei and IgG4 immunostaining, yielded negative results, with an IgG4/IgG ratio of much less than 40% (Figure 3E, 3F).

## Discussion

SANT is a rare benign vascular lesion of the spleen. This entity was first described by Krishnan et al. in 1993 and was later formally designated SANT by Martel et al. in 2004 [7]. To date, at least 258 SANT cases have been reported in the literature [1]. Despite its benign nature, the preoperative diagnosis remains challenging owing to the nonspecific clinical presentation and overlapping radiological features of malignant splenic tumors [3,4].

This case report highlights the diagnostic challenges and unique clinicopathological features of this disease. Unlike the typical middle-aged female predominance reported in the literature, where female cases account for approximately 60% of cases [6], our patient was a young male, suggesting a more balanced sex distribution than previously thought. The presentation of chronic left upper quadrant pain is also noteworthy, as most SANT cases are incidentally discovered during imaging for unrelated conditions [6]. This atypical symptomatic presentation underscores the importance of maintaining clinical suspicion in patients with persistent unexplained abdominal pain.

Radiologically, the lesion exhibited peripheral enhancement on CT, raising initial concerns for malignancy, consistent with prior reports [1]. However, a definitive diagnosis relies on a histopathological examination, which confirms the characteristic features of SANT, including concentric fibrosclerotic stroma, angiomatoid nodules, and the presence of slit-like vascular spaces filled with red blood cells [1]. Immunohistochemistry further supported the diagnosis, revealing the hallmark vascular triad (CD34⁺/CD31⁺/CD8⁻ capillaries, CD8⁺/CD31⁺/CD34⁻ sinusoids, and CD31⁺ venules) mimicking splenic red pulp architecture [7]. In addition, α-SMA positivity supports the presence of stromal myofibroblastic proliferation, a frequent finding in SANT [8]. Although EBV and IgG4-related diseases have been linked to various splenic lesions, no evidence of their involvement was found in the present patient [10]. 

Despite the increased awareness of SANT, its pathogenesis, natural history, and risk of recurrence remain poorly understood. There is ongoing debate regarding whether SANT represents a reactive, inflammatory, or neoplastic process [2,7-9]. Notably, an earlier study proposed that SANT is likely a polyclonal, reactive lesion distinct from the inflammatory pseudotumor-like features characteristic of IgG4-related sclerosing disease [11]. To further clarify the biological behavior of this lesion, additional molecular studies and long-term follow-up data are warranted. Furthermore, the development of reliable noninvasive diagnostic markers may help reduce the need for splenectomy in asymptomatic patients. This case highlights the diagnostic complexity of SANT and emphasizes the importance of integrating the clinical, radiological, histopathological, and immunohistochemical findings. Continued research is essential to improve preoperative diagnostic accuracy and elucidate the pathophysiology of this rare splenic lesion [6-9].

## Conclusions

SANT of the spleen is a rare benign lesion that often presents with nonspecific clinical and radiological features, making a preoperative diagnosis difficult. This case highlights the crucial role of a histopathological examination, in conjunction with immunohistochemistry, in distinguishing SANT from other benign and malignant splenic tumors. Clinicians should consider SANT in the differential diagnosis of solitary splenic masses, especially in patients with chronic unexplained abdominal pain. An accurate histopathological diagnosis plays a key role in establishing a definitive diagnosis and guiding subsequent treatment decisions. Additional molecular studies are warranted to better understand its pathogenesis and its potential clinical significance.

## References

[1] Alemu S, Mulatu B, Kedir A, Minka M, Tesfaye W, Reta Demissie W (2025). A rare case of sclerosing angiomatoid nodular transformation of spleen: A case report. Int J Surg Case Rep.

[2] Weinreb I, Bailey D, Battaglia D, Kennedy M, Perez-Ordoñez B (2007). CD30 and Epstein-Barr virus RNA expression in sclerosing angiomatoid nodular transformation of spleen. Virchows Arch.

[3] Vigorito R, Scaramuzza D, Pellegrinelli A, Marchianò A (2019). Sclerosing angiomatoid nodular transformation (SANT) of the spleen: A case report on CT and MRI. BJR Case Rep.

[4] Ma J, Zhang W, Wang L, Zhu Z, Wang J, Zhang J, Yang X (2019). Imaging features of sclerosing angiomatoid nodular transformation in spleen. J Comput Assist Tomogr.

[5] Liao J, Wang Z, Li Q (2021). CT and MRI features of sclerosing angiomatoid nodular transformation of the spleen: A report of 18 patients with pathologic correlation. Diagn Interv Imaging.

[6] Falk GA, Nooli NP, Morris-Stiff G, Plesec TP, Rosenblatt S (2012). Sclerosing angiomatoid nodular transformation (SANT) of the spleen: Case report and review of the literature. Int J Surg Case Rep.

[7] Martel M, Cheuk W, Lombardi L, Lifschitz-Mercer B, Chan JK, Rosai J (2004). Sclerosing angiomatoid nodular transformation (SANT): Report of 25 cases of a distinctive benign splenic lesion. Am J Surg Pathol.

[8] Kuo TT, Chen TC, Lee LY (2009). Sclerosing angiomatoid nodular transformation of the spleen (SANT): Clinicopathological study of 10 cases with or without abdominal disseminated calcifying fibrous tumors, and the presence of a significant number of IgG4+ plasma cells. Pathol Int.

[9] Raja F, Kumar V, Moll E, Hammad A, Ayub S (2023). Sclerosing angiomatoid nodular transformation of the spleen: A report of rare case and literature review. Cureus.

[10] Deshpande V, Zen Y, Chan JK (2012). Consensus statement on the pathology of IgG4-related disease. Mod Pathol.

[11] Chang KC, Lee JC, Wang YC (2016). Polyclonality in sclerosing angiomatoid nodular transformation of the spleen. Am J Surg Pathol.

